# Impact of Chaperone-Mediated Autophagy in Brain Aging: Neurodegenerative Diseases and Glioblastoma

**DOI:** 10.3389/fnagi.2020.630743

**Published:** 2021-01-28

**Authors:** Jaione Auzmendi-Iriarte, Ander Matheu

**Affiliations:** ^1^Cellular Oncology Group, Biodonostia Health Research Institute, San Sebastian, Spain; ^2^CIBER de Fragilidad y Envejecimiento Saludable (CIBERfes), Madrid, Spain; ^3^IKERBASQUE, Basque Foundation, Bilbao, Spain

**Keywords:** CMA, LAMP2, physiological aging, neurodegenerative diseases, glioblastoma

## Abstract

Brain aging is characterized by a time-dependent decline of tissue integrity and function, and it is a major risk for neurodegenerative diseases and brain cancer. Chaperone-mediated autophagy (CMA) is a selective form of autophagy specialized in protein degradation, which is based on the individual translocation of a cargo protein through the lysosomal membrane. Regulation of processes such as proteostasis, cellular energetics, or immune system activity has been associated with CMA, indicating its pivotal role in tissue homeostasis. Since first studies associating Parkinson’s disease (PD) to CMA dysfunction, increasing evidence points out that CMA is altered in both physiological and pathological brain aging. In this review article, we summarize the current knowledge regarding the impact of CMA during aging in brain physiopathology, highlighting the role of CMA in neurodegenerative diseases and glioblastoma, the most common and aggressive brain tumor in adults.

## Introduction to Autophagy

The term “autophagy” comes from the Ancient Greek meaning “self-eating,” which refers to the catabolic processes that use the cell to recycle its own constituents within the lysosome (Yang and Klionsky, 2010; Boya et al., 2013). The first discoveries were based on the bulk trapping of the cargo in double-membrane autophagosomes that fuse with lysosomes, which afterward was named “macroautophagy” (De Duve, 1963). Further studies demonstrated also the existence of another type of autophagy named “microautophagy,” which involves the uptake of soluble or membrane-bound material directly into the lysosome by invagination (De Duve and Wattiaux, 1966; Marzella et al., 1981). However, it was not until Dice et al. (1986) described a protein-selective form of autophagy, identifying a pentapeptide region on ribonuclease A required for its enhanced degradation during serum deprivation. This form of autophagy, nowadays known as “chaperone-mediated autophagy” (CMA), was at that time the unique selective form of autophagy. Since then, selective forms of macroautophagy and microautophagy have been described (Klionsky et al., 2016), with a special interest in “chaperone-assisted selective autophagy” (CASA), which participates in the selective trapping of ubiquitin-positive protein aggregates *via* autophagosomes (Arndt et al., 2010) and “endosomal-microautophagy,” in which cytosolic proteins enter endosomal compartments inside vesicles generated at the surface of the late endosomes (Sahu et al., 2011).

However, among all forms of autophagy, CMA is still the only process that enables the direct translocation of an individual cargo protein across the lysosomal membrane for its degradation (Kaushik and Cuervo, 2018). Thus, herein we will review the principal components and mechanisms involved in CMA and highlight its impact on physiological aging of the brain, with special emphasis on neurodegenerative disorders and glioblastoma.

## Cma Process

### Mechanism of Action

**Figure 1:**
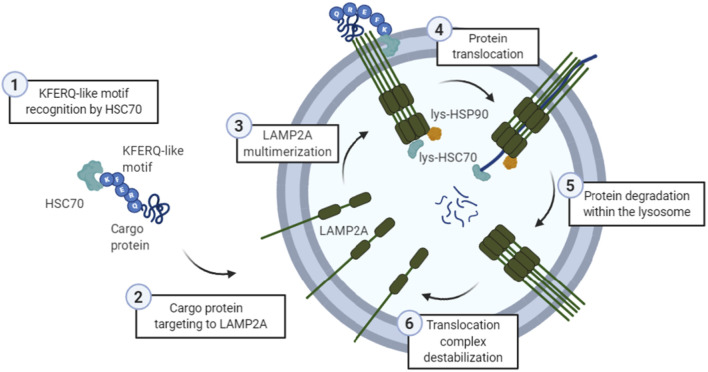
General scheme of the chaperone-mediated autophagy (CMA) process. (1) The KFERQ-like motif of a cargo protein is detected by HSC70, (2) which is the one targeting it into the lysosomal membrane protein lysosome-associated membrane protein type 2A (LAMP2A). Then, (3) LAMP2A monomers are assembled into multimeric structures, forming the translocation complex. Once this structure is correctly formed, (4) protein is translocated through the lysosomal membrane, while lysosomal HSC70 (lys-HSC70) pulls it into the lumen and avoid the return out of the membrane. Lysosomal HSP90 (lys-HSP90) is important in LAMP2A stabilization since it masks lysosomal protease binding sequences, and thus, prevents its degradation during this transition. Finally, (5) protein is degraded inside the lysosome, and (6) translocation complex is disassembled.

Biochemical and genetic approaches have characterized CMA as a rigorous process where the cargo protein must overcome several stages to reach proper lysosomal degradation (Figure 1).

#### Cargo Protein Selection

For a specific protein to be degraded *via* CMA, it first has to bind to the heat shock cognate 71 kDa protein (HSC70), a chaperone that is going to target it to the lysosomal membrane (Dice, 1990). For the recognition of this cargo protein, a “KFERQ” motif is needed (Kaushik and Cuervo, 2018), which comprises a sequence of amino acids with specific charge and hydrophobicity. Overall, it is known that approximately 40% of the mammalian proteome could contain this canonical motif (Kaushik and Cuervo, 2018). However, post-translational modifications, such as phosphorylation, acetylation, or even ubiquitination, within incomplete motifs can confer specific properties, recreating a complete KFERQ-like motif (Kaushik and Cuervo, 2018). Not only inside the motif, but post-translational modifications outside the motif can even expose or mask an existing one, by conformational changes of the protein (Ferreira et al., 2015; Kaushik and Cuervo, 2018). Thus, CMA substrates can vary substantially. In this line, a free web-based resource called “KFERQ finder” has been developed, which directly identifies KFERQ-like motifs in any protein sequence (Kirchner et al., 2019).

#### HSC70 Binding and Targeting to Lysosomal Membrane

Hidden KFERQ motifs become accessible to HSC70 after: (I) protein conformational changes; (II) multiprotein complex disassembly; or (III) releasing from the subcellular membranes (Cuervo and Wong, 2014). Once bound to the chaperone, the substrate is delivered to the surface of the lysosome where it interacts with the cytosolic tail of the lysosome-associated membrane protein type 2A (LAMP2A; Cuervo and Dice, 1996). It is important to highlight that HSC70’s role in autophagy has been assigned not only to CMA but also to CASA (Arndt et al., 2010) and endosomal-microautophagy (Sahu et al., 2011).

#### LAMP2A Targeting and Substrate Translocation

LAMP2A is one of the three splicing variants of the *LAMP2* gene, which contains a cytosolic tail that differs from the other isoforms and is crucial for CMA to occur (Cuervo and Dice, 2000b). Substrates can bind to LAMP2A in a folded state, but they must be unfolded to be translocated to the lumen of the lysosome (Salvador et al., 2000). In this aspect, HSC70 and other co-chaperones located in the membrane of the lysosome are thought to be mediating this process (Cuervo and Wong, 2014; Rout et al., 2014). During this step, LAMP2A monomers are multimerized, while the heat shock cognate 90 kDa (HSC90) chaperone stabilizes the complex from the luminal part, hiding the protease-sensitive regions of LAMP2A (Bandyopadhyay et al., 2008). When the translocation complex, which comprises 700 kDa, is formed and the cargo protein is unfolded, the substrates enter the lumen of the lysosome. Moreover, there is a form of HSC70 located within the lysosome (lys-HSC70), which reinforces the translocation of the substrate pulling it and preventing its return into the cytosol (Agarraberes and Dice, 2001). A recently published bioinformatic study demonstrated that several domains of the *LAMP2* gene present positive selective pressure in placental mammals, giving new insights regarding the molecular evolution of the key protein of CMA (Jalali and Parvaz, 2020). Future work should define the functional impact of those selective substitutions in CMA and autophagy, particularly in the brain. Nevertheless, dependence on LAMP2A is the best criteria to determine whether a specific protein is degraded by CMA (Kaushik and Cuervo, 2018).

### Regulation of CMA Function

**Figure 2:**
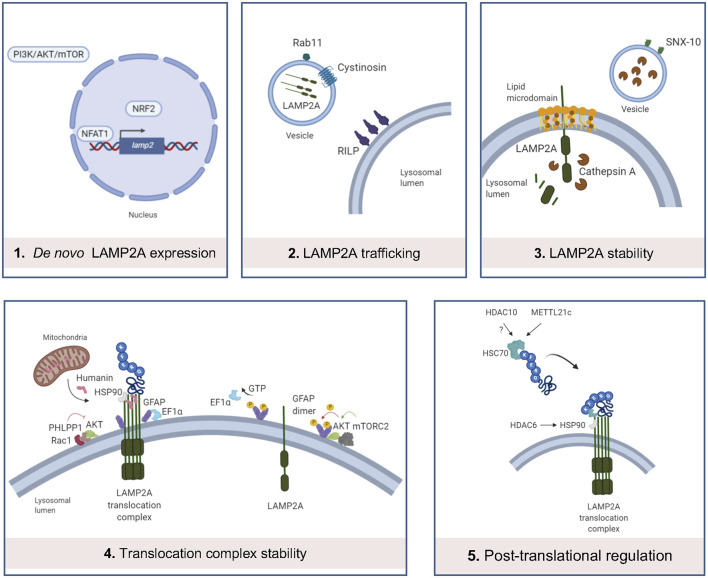
Main levels of regulation of CMA. (1) *LAMP2A* *de novo* expression can be regulated by the binding of NFAT1 to the promoter of *LAMP2* gene, binding of NRF2 and PI3K/AKT/mTOR pathway; (2) LAMP2A trafficking into the lysosomal membrane is altered by cystinosin and Rab11 and RILP protein dysfunctions; (3) in resting conditions, LAMP2A monomers are located in lipid microdomains, where LAMP2A is more sensitive to cathepsin A-mediated degradation. SNX-10 deficiency upregulates LAMP2A expression, probably by dysregulation of cathepsin A trafficking into the lysosomes; (4) Rac1/PHLPP1 complex inhibits Akt, enabling glial fibrillary acidic protein (GFAP)-mediated translocation complex stabilization. On the contrary, this complex is destabilized by GTP-mediated release of Elongation factor 1α (EF1α) from the lysosomal membrane and mTORC2/Akt phosphorylation of GFAP, promoting GFAP self-association. Humanin, a mitochondria-associated peptide, interacts with HSP90 and stabilizes the binding of this chaperone to CMA substrates; (5) recent studies demonstrate that METT21c and potentially HDAC10 regulate HSC70 action by post-transcriptional regulation. HDAC6 regulates HSP90 acetylation and activity.

The CMA process is tightly regulated and this facilitates the maintenance of cell and organism homeostasis. As LAMP2A is the main effector of CMA, its expression, trafficking, and stabilization are key aspects for CMA to occur. Next, we summarize the regulators of these processes and we provide recent data regarding epigenetic regulation of chaperones and CMA (Figure 2).

#### *De novo* LAMP2A Synthesis

The first transcription factor identified in the regulation of CMA was the Nuclear Factor of Activated T Cells 2 (NFATC2), also known as NFAT1, which, after activation by calcium-calcineurin, is recruited to the *Lamp2* promoter to induce its expression, mediating the response to reactive oxygen species (ROS) in activated murine T cells (Valdor et al., 2014). Together with NFAT1, nuclear factor erythroid 2-related factor 2 (NRF2) transcription factor has been also described as a modulator of *LAMP2A* expression and consequently, CMA activity in both human and mouse cell types, as the *LAMP2* gene presents binding sequences for NRF2 (Pajares et al., 2018). Interestingly, the study of *de novo* *LAMP2A* expression modulation has been extended from specific transcription factors to other signaling pathways. Among them, the PI3K/AKT/mTOR signaling pathway should be pointed out, since it has been described as a negative regulator of *LAMP2A* and *HSC70* expression (Li et al., 2017b), but also macroautophagy activity (Heras-Sandoval et al., 2014). However, the detailed mechanism of how this pathway modulates *LAMP2A* expression remains to be elucidated.

#### LAMP2A Trafficking to the Lysosome

Different subcellular distributions of LAMP2 splicing variants have been assigned largely by the COOH-terminal amino acid residue (Gough and Fambrough, 1997). Besides this regulatory level, other key effectors in LAMP2A trafficking have been discovered in a cystinosis model. Herein, cystinosin (CTNS) has been associated with the regulation of LAMP2A trafficking, as Ctns−/− cellular model presents reduced co-localization of lysosomal membrane protein LAMP1 and LAMP2A, and a significantly increased number of LAMP2A-positive vesicles with motility restriction (Zhang et al., 2017). Indeed, up-regulation of Rab11 or Rab7/RILP leads to the correction of the LAMP2A mislocalization in Ctns−/− cells, which shows their regulatory role in LAMP2A trafficking, at least, in this model (Zhang et al., 2017). Future studies should evaluate the role of CTNS, Rab11, and RILP in LAMP2A trafficking under physiological conditions.

#### LAMP2A Stability in the Lysosomal Membrane

Levels of LAMP2A at the lysosomal membrane are mainly modulated by changes in its distribution and half-life. A percentage of LAMP2A is located in lipid-enriched membrane microdomains during resting conditions, and its organization into multimeric complexes for substrate translocation only occurs outside these regions (Kaushik et al., 2006). Loading of lysosomes with cholesterol significantly reduces CMA, mainly due to the increased susceptibility of LAMP2A to be cleaved by proteases such as cathepsin A and posterior degradation in the lysosomal lumen (Cuervo et al., 2003). In this line, sorting nexin (SNX)-10 deficiency upregulates LAMP2A and so, CMA activation, probably due to a dysregulation of cathepsin A trafficking into lysosomes and thus, protecting LAMP2A from its degradation (You et al., 2018).

#### Translocation Complex Stability

GTP presents an inhibitory effect on CMA by altering the stability of the translocation complex, mediated by glial fibrillary acidic protein (GFAP) and elongation factor 1α (EF1α), in mouse and rat models (Bandyopadhyay et al., 2010). In the absence of GTP stimulus, GFAP stabilizes the multimeric translocation complex, whereas GTP-mediated release of EF1α from the membrane of the lysosome promotes self-association of GFAP, reducing the stabilization of the complex and consequently diminishing CMA activity (Bandyopadhyay et al., 2010). An additional study shows that the interaction between Akt and mTORC2/PHLPP1 also controls the dynamics of the assembly and disassembly of the translocation complex (Arias et al., 2015). In particular, when associated with the lysosomal membrane, the phosphatase PHLPP1 and the kinase mTORC2 regulate the phosphorylation state of Akt, modulating thus the stability of the translocation complex (Arias et al., 2015). Moreover, Humanin, a mitochondria-associated peptide, interacts with HSP90, and stabilizes the binding of this chaperone to CMA substrates (Gong et al., 2018).

#### Post-translational Regulation of HSC Chaperones

Several studies have recently shown new functions of deacetylase and methyltransferase enzymes on the activity of chaperones within CMA. An initial study revealed that histone deacetylase 6 (HDAC6) regulates HSP90 acetylation, thus controlling its interaction with LAMP2A and HSC70, under the hypoxia-ischemia-stress induced by acute spinal cord injury (Su et al., 2016). Other studies showed that histone deacetylase 10 (HDAC10) deacetylates HSC70 (Oehme et al., 2013) and that HDAC10 knock-out accumulates LAMP2A positive lysosomes around the nucleus, activating CMA (Obayashi et al., 2020). In the same line, an additional study described that the newly classified methyltransferase-like 21c (METTL21c) trimethylates HSP70 at Lys-561 to enhance its stability, inducing its function in CMA in mice (Wang et al., 2019a). All these recent data exhibit the potential that epigenetic enzymes may have in CMA regulation.

### Crosstalk With Other Types of Protein Degradation Systems

Although three isoforms of the *LAMP2* gene present a common luminal domain, their different cytosolic and transmembrane domains (Cuervo and Dice, 2000b) give them unique properties that hamper compensation of all functions; while LAMP2A is the only isoform that can work in CMA, LAMP2B is associated to macroautophagy (Nishino et al., 2000; Chi et al., 2019) and LAMP2C to a selective type of autophagy called “RNautophagy” (Fujiwara et al., 2013).

Compensatory mechanisms between different proteolytic systems within the cell have been widely described for the correct proteostasis of the tissue (Park and Cuervo, 2013; Wu et al., 2015; Dong and Cui, 2018). Indeed, a recent proteome-wide study identified a globally harmonized rhythm for basal macroautophagy, CMA, and proteasomal activity for proteolysis during the daytime (Ryzhikov et al., 2019).

Regarding macroautophagy, it can be upregulated in CMA-defective conditions to maintain normal rates of long-lived protein degradation, although is unable to compensate for all CMA features (Massey et al., 2006). Supporting this original evidence, a study showed that the upregulation of CMA mediated by the stress caused by hepatitis C, caused selective degradation of beclin1, thus impairing autophagosome-endosome fusion, a key step in the macroautophagic process (Aydin et al., 2018). In the same way, the blockage of macroautophagy leads to upregulation of CMA (Kaushik et al., 2008), which has been associated with resistance to death induced by oxidative stress from menadione or UV light (Singh and Czaja, 2008). Another evidence that supports this mutual regulation of macroautophagy and CMA is that protein kinase Cα (PKCα) mediated phosphorylation of ULK1, on the one hand, prevents autolysosome formation, but on the other hand, enhances its interaction with HSC70 and increases its degradation through CMA (Wang et al., 2018a). However, macroautophagy and CMA can also work together in a coordinated way, as it is demonstrated in a study where, to occur hydrolysis of lipid droplets (LD) during starvation, CMA degradation of PLIN2 and PLIN3 was enhanced, concurrent with elevated levels of macroautophagy proteins on LDs (Kaushik and Cuervo, 2015). The ubiquitin-proteasome system (UPS) is the main non-lysosomal degradative pathway for ubiquitinated proteins (Pandey et al., 2007). Notably, it has been described as the upregulation of CMA (Koga et al., 2011b) and an accumulation of proteasomal subunits in lysosomes in response to inhibition of the proteasome (Cuervo et al., 1995; Goebel et al., 2020). On the contrary, in the first stages of CMA blockage, the activity of both macroautophagy and UPS has been identified to be impaired, precipitating the intracellular accumulation of altered components and making the cell unable to eliminate them even after the subsequent restoration of normal UPS and activation of macroautophagy (Massey et al., 2008).

Interestingly, some of these mechanisms have been described in the brain. Firstly, ubiquilins, highly conserved ubiquitin-binding proteins, have been recently described as integrators of UPS and lysosomal degradation for neuronal maintenance, *via* mTOR signaling (Šentürk et al., 2019). Indeed, a previous study demonstrated that ubiquilin is present in autophagosomes from brain tissue of mice and that it is degraded during both macroautophagy and CMA (Rothenberg et al., 2010). Not only ubiquilins but also HDAC6, which interacts with polyubiquitinated proteins, has been identified as an essential mechanistic link between UPS and autophagy in a neurodegenerative model (Pandey et al., 2007).

## Cma During Physiological Aging

The important regulatory role of CMA highlights its relevance in organ functionality and tissue maintenance. Indeed, a functional decline of CMA during physiological aging has been found in the last decades (Cuervo and Dice, 2000a; Zhou et al., 2017). Interestingly, it has been described that not its transcriptional rate, but the stability of LAMP2A within the lysosomal membrane is altered with age (Kiffin et al., 2007), which indeed can be further degenerated after chronic exposure to a high-fat diet (Rodriguez-Navarro et al., 2012). On the contrary, mice with an inducible exogenous extra copy of *LAMP2A* in the liver displayed reduced damaged proteins and improved organ function (Zhang and Cuervo, 2008), reinforcing its action on physiological aging. In line with this, resveratrol, an agent with anti-aging activity, upregulates *LAMP2* in hepatocytes (Lee et al., 2019). Liver lysosomes from long-lived both Pou1f1/Pit1 mutant (Snell) mice and growth hormone receptor (ghr) knockout mice also present increased CMA substrate uptake activity (Endicott et al., 2020), further associating the beneficial impact of maintaining CMA with healthspan and longevity.

## Cma During Physiological Aging in The Brain

**Figure 3:**
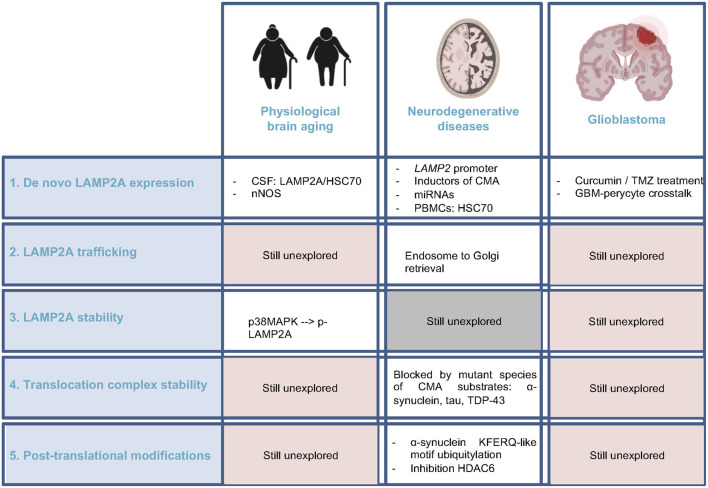
Current knowledge regarding CMA regulators alteration in brain aging, neurodegenerative diseases, and glioblastoma. Comparison of the pieces of evidence among these three physiopathological conditions of the brain, highlighting the unexplored fields. CSF, cerebrospinal fluid; nNOS, neuronal nitric oxide synthase; PBMC, peripheral blood mononuclear cell.

The impact of autophagy and specifically CMA, in physiological brain aging remains largely unknown, although some evidence is supporting the downregulation of relevant proteins involved in it (Table 1, Figure 3). Almost all of the studies published so far remain correlative, suggesting an impaired regulation of *de novo* LAMP2A synthesis. Among them, a study showed a significant decline of HSC70 (Loeffler et al., 2016) and little changes in LAMP2 (Loeffler et al., 2018) concentration in cerebrospinal fluid (CSF). Interestingly, neuronal nitric oxide synthase (nNOS) has been associated with neuronal aging in rodents, causing LAMP2A reduction and consequent accumulation of CMA substrates (Valek et al., 2019). In the same way, CMA impairment is detrimental for neuronal viability *in vivo* (Xilouri et al., 2016) and identified PARK7/DJ-1 and DPYSL2/CRMP-2 as the most significantly altered proteins upon LAMP2A downregulation in primary rat cortical neurons (Brekk et al., 2019). Interestingly, PARK7/DJ-1 contains overlapping canonical and acetylation-generated KFERQ-like motifs 10 residues upstream of ^106^C shown to be key for the activation of this protein by oxidative stress. It has been postulated that the oxidative status of ^106^C may change the exposure of the motif to HSC70 binding and subsequent CMA-mediated degradation, or prevent it by ubiquitylation (Kirchner et al., 2019). As oxidative stress has been associated with aging, this evidence makes it attractive to propose that post-translational modifications in KFERQ-like motifs of CMA substrates could have an impact on the aging phenotype.

**Table 1:** Main studies revealing chaperone-mediated autophagy (CMA) downregulation in brain homeostasis and aging.

Molecule	Role in CMA	Finding	Model of study	Reference
LAMP2A	Key effector	Its reduction induces progressive loss of nigral dopaminergic neurons, increased astro-microgliosis and motor deficits.	Rat substrantia nigra *in vivo*	Xilouri et al. (2016)
		Alters the expression of PARK7/DJ-1 and DPYSL2/CRMP-2 proteins, with measurable effects in neuronal homeostasis and phenotype.	Primary rat cortical neurons	Brekk et al. (2019)
*LAMP2*	Gene coding *LAMP2A*	Downregulation in the expression in aged patients.	Human CSF	Loeffler et al. (2018)
HSPA8	Effector	Downregulation in the expression in aged patients.	Human CSF	Loeffler et al. (2016)
nNOS	Regulator	Associated to neuronal aging, causes an accumulation of CMA substrates and loss of LAMP2A.	Human neuroblastoma cell line	Valek et al. (2019)

*CFS, cerebrospinal fluid*.

Studies regarding altered regulation of LAMP2A stability in lysosomal membrane could also be associated with brain aging. In this line, p38MAPK, which phosphorylates LAMP2A causing its lysosomal membrane accumulation and active conformational change (Li et al., 2017a), has been recently identified as a regulator of neuronal and neural stem cell (NSC) activity during brain aging (Moreno-Cugnon et al., 2019, 2020), which support the idea that alterations in CMA regulation may be associated to brain aging.

Besides, a decline in the integrity of the blood-brain barrier (BBB) is a major feature of brain aging, and it has been speculated that downregulation of endothelial annexin A1, a CMA regulator (Liu et al., 2018), maybe partially responsible for the aged BBB phenotype (McArthur et al., 2016).

Considering all these ideas, it is reasonable to suggest that CMA activity could decrease in brain physiological aging, although still very preliminary (Figure 3). Thus, future studies should be directed to the elucidation of this hypothesis, focusing on different cell types among the brain and the crosstalk with UPS or macroautophagy, with a special interest in *in vivo* models.

## Physiological Roles of Cma in The Brain

Protein degradation by CMA regulates diverse cellular processes so that its balanced activity is crucial, especially in the brain. Several studies have linked the onset of aging and age-associated neurodegenerative diseases to the downregulation of CMA (Cuervo and Dice, 2000a; Alfaro et al., 2018; Loeffler, 2019). Regarding malignant transformation, it has been described that CMA presents anti-oncogenic effects in untransformed healthy cells (Arias and Cuervo, 2020) suggesting that brain aging-associated CMA decline could be a risk factor for brain tumorigenesis. Once the damage is accumulated, tumor cells activate CMA constitutively, promoting brain cancer progression (Valdor et al., 2019).

**Figure 4:**
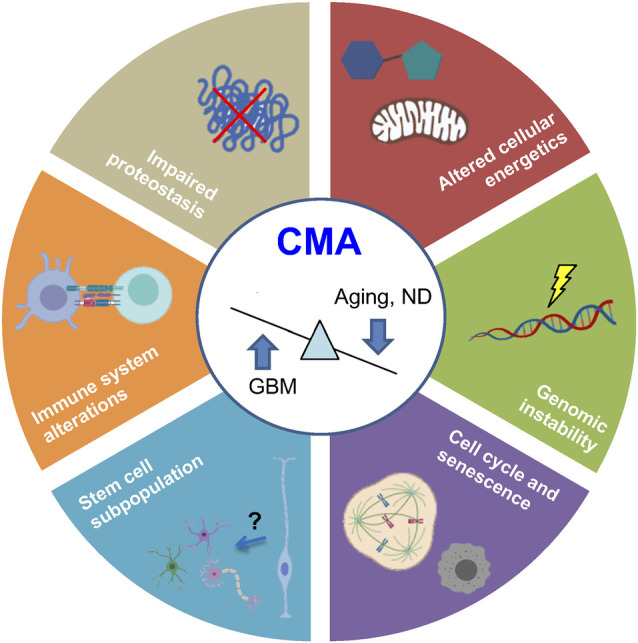
Common hallmarks of aging and cancer regulated by CMA. Recent evidence demonstrates that physiological aging and ND are associated with CMA downregulation, whereas glioblastoma (GBM) presents an upregulated activity. Processes regulated by CMA are represented in the figure. ND, neurodegenerative diseases.

It is widely accepted that both aging and cancer are two manifestations of the accumulation of cellular and DNA damage (López-Otín et al., 2013) so that CMA acts as a cell survival mechanism. In particular, CMA participates in the regulation of various common hallmarks of aging (López-Otín et al., 2013) and cancer (Hanahan and Weinberg, 2011), where, the existing data could suggest that, while brain cancer cells use CMA for survival, age-dependent CMA decline prevents recovery and restoration of damaged tissues (Figure 4).

### Altered Proteostasis

The most characterized role of CMA is the maintenance of a correct proteostasis, which has been widely described to be impaired in aging and neurodegenerative diseases (López-Otín et al., 2013) and must be maintained in cancer cells to survive (Yang et al., 2019). In this sense, oxidative stress (Law et al., 2016; Issa et al., 2018) and hypoxic stress (Dohi et al., 2012) have been described to activate CMA to reduce protein damage in the brain.

### Impaired Cellular Energetics and Metabolism

Besides quality control, there is currently evidence that supports that protein damage is not a requirement for CMA targeting (Kaushik and Cuervo, 2018). Cellular energetics, another common hallmark of aging and cancer, has been linked to CMA since its discovery when it was described that starvation-induced activation (Dice et al., 1986). This evidence was associated for a long time with a mechanism in which a pool of free amino acids was needed. However, recent studies show that the role of enzymes degraded by CMA in metabolism could be responsible for cellular energetic changes (Tasset and Cuervo, 2016). Interestingly, lipid and carbohydrate metabolic alterations caused by altered CMA have been described to be associated with physiological aging of the brain (Hallett et al., 2018) and neurodegenerative diseases (Alfaro et al., 2018). Not only that, but macrophage-specific LAMP2A deficient mice also exhibited significant intracellular lipid accumulation (Qiao et al., 2020), extending the impact of CMA to immune cells, which has been linked to brain homeostasis and disease (Li and Barres, 2018; Dulken et al., 2019). Regarding amino acid metabolism, the accumulation of cystine within the lysosome occurred in cystinosis, a disorder that affects also the brain (Jonas et al., 1987), which is associated with CMA activity (Zhang et al., 2017).

### Augmented Genomic Instability

Genotoxic insults together with oxidative stress and starvation are the main stimulus which activates CMA (Park et al., 2015). In this regard, CHK1 participates in genome quality control, and it has been associated with physiological neuronal differentiation (Oshikawa et al., 2017) and resistance of glioblastoma cells to chemo/radiotherapy (Patties et al., 2019). Interestingly, CHK1 has been described as a CMA substrate, as it presents various KFERQ-like motifs in different domains (Kirchner et al., 2019). Indeed, phosphorylation of ^345^S, separated only by four amino acids from the canonical KFERQ-motif, produces conformational changes, altering HSC70 recognition, and subsequent degradation *via* CMA (Kirchner et al., 2019). The abnormal nuclear accumulation of phosphorylated CHK1 after a genotoxic insult in CMA-deficient condition alters proteins involved in cell cycle regulation and DNA repair, inducing levels of DNA damage marker p-γH2AX (Park et al., 2015). Indeed, ubiquitylation events in the putative motif of the catalytic region may also modulate the degradation of still inactive CHK1 (Kirchner et al., 2019), suggesting that many post-translational modifications within KFERQ-like motifs may modulate CHK1 degradation *via* CMA and consequently, regulate DNA repair response.

### Cell Cycle Dysregulation and Senescence

Some studies describe the impact of CMA on cell cycle and senescence. On the one hand, tumor suppressor proteins such as p73 (Nguyen et al., 2020) or mutant p53 (Vakifahmetoglu-Norberg et al., 2013), and transcription factors MEF2A (Zhang et al., 2014) and MEF2D (Yang et al., 2009), previously associated to embryonic and adult neurogenesis processes, have been denominated as CMA substrates. Besides, it has been proposed that CMA dysfunction during aging promotes cellular senescence (Moreno-Blas et al., 2018). In contrast, CMA also participates in ferroptosis (Wu et al., 2019b), a specific necrotic cell death that has been linked to diverse age-associated brain diseases (Weiland et al., 2019).

### Changes in Stem Cell Subpopulation

Alterations in stem cell subpopulations have been widely described in brain pathologies, with an age-dependent physiological decline of the NSC pool (Schultz and Sinclair, 2016) and the presence of malignant cancer stem cells in glioblastoma (Garros-Regulez et al., 2016; Arrizabalaga et al., 2017). Interestingly, old quiescent NSCs display defects in their lysosomal activity, with an increased accumulation of protein aggregates and reduced ability to be activated (Leeman et al., 2018). Many studies have focused on macroautophagy (Revuelta and Matheu, 2017; Sotthibundhu et al., 2018; Ryskalin et al., 2019), but the impact of CMA in NSC maintenance remains mostly elusive. To date, some studies could give insights about the possible link between stem cell regulation and the CMA process. On the one hand, retinoic acid has been associated with neural and astroglial differentiation during development (Hádinger et al., 2009; Tan et al., 2015), adulthood (Jacobs et al., 2006; Mishra et al., 2018), and glioblastoma pathogenesis (Ying et al., 2011) and this signaling pathway inhibits CMA (Anguiano et al., 2013). In the same way, hypoxia promotes NSC proliferation *via* Hypoxia-inducible factor 1-α (HIF1α; Hubbi and Semenza, 2015), which is targeted for lysosomal degradation by CMA (Ferreira et al., 2013; Hubbi et al., 2013). Moreover, mice with an extra copy of Arf/p53 display delayed exhaustion of NSCs and enhanced cognitive activity (Carrasco-Garcia et al., 2015, 2017). However, further research would be needed to test these associations and get direct evidence regarding CMA impact, if any, in stem cell maintenance.

### Immune System Alterations

The first evidence that linked CMA to the immune system was that LAMP2A participates in the display of cytoplasmic epitopes *via* class II molecules in antigen-presenting cells (Zhou et al., 2005). Further research has shown a positive association between CMA and activation of T cells (Valdor et al., 2014) and tumor-associated macrophages in hepatocellular carcinoma (Guo et al., 2017) and breast cancer (Wang et al., 2019b). However, very little is known regarding the physiological role of CMA in this organ, although several studies have described the role of other types of autophagy in the brain’s immune system (Plaza-Zabala et al., 2017; Molina et al., 2019). Thus, CMA degrades IKKβ and thereby reduces TNFα expression in microglia (Liu et al., 2018) and CMA inhibition contributes to mesenchymal stem cell immunosuppressive function (Zhang et al., 2020). Further studies are needed to elucidate the role of CMA in the brain’s immune system and its crosstalk with other cell types.

## Cma in Neurodegenerative Diseases

It was neurodegenerative Parkinson’s disease (PD) the first disorder associated with CMA (Cuervo et al., 2004). Since then, research has been extended to other prevalent neurodegenerative disorders, demonstrating a reduction in CMA activity in most of them (Table 2, Figure 3).

**Table 2:** Main studies revealing CMA downregulation in neurodegenerative diseases.

Disease type	Role in CMA	Molecule	Finding	Model of study	Reference
PD	Gene coding *LAMP2A*	*LAMP2*	Study of *LAMP2* promoter variants and its association with sporadic PD	Human peripheral leukocytes genomic DNA	Pang et al. (2012)
			Expression diminishment in female LRRK2 PD	Human CFS	Klaver et al. (2018)
	Effector	LAMP2A	Reduction in the expression of LAMP2A and Hsc70 in PD patients	Substantia nigra pars compacta and amygdala of PD and control brains	Alvarez-Erviti et al. (2010)
			Several miRNA regulate negatively LAMP2A in PD	Neuroblastoma cell line and human PD brains	Alvarez-Erviti et al. (2013)
			Impaired endosome-to-golgi retrieval of LAMP2A accumulate α-syn	VPS35 mutant mice	Tang et al. (2015)
			Overexpression of LAMP2A causes decreased α-syninduced dopaminergic neurodegeneration	Human neuroblastoma cells, rat primary cortical neurons *in vitro*, and nigral dopaminergic neurons *in vivo*	Xilouri et al. (2013)
		Hsc70	Reduction of the expresion in sporadic PD	Human PBMCs	Sala et al. (2014)
	CMA substrate	α-syn	First evidence of α-syn degradation by CMA. Effect of mutant forms in CMA perforrmance	Rat ventral midbrain neuronal cultures	Cuervo et al. (2004)
			*in vivo* degradation of α-syn under normal and enhanced protein burden conditions	C57BL/6 and transgenic mice overexpressing wild-type mouse α-syn	Mak et al. (2010)
			Dopamine-modified α-syn effect in CMA	Mouse ventral medial neuron culture, neuroblastoma cell line	Martinez-Vicente et al. (2008)
		LRRK2	First evidence of LRRK2 degradation by CMA. Effect of G2019S mutant form in CMA perforrmance	BAC transgenic for WT and mutant LRRK2, patient-derived iPSCs, human brain tissue (DMV)	Orenstein et al. (2013)
	Regulator	UCH-L1	UCH-L1 interaction with LAMP2A, Hsc70 and Hsp90. Effect of I93M UCH-L1 mutation	Human and murine fibroblasts	Kabuta et al. (2008)
		Nrf2	Nrf2 in astrocytes delays CMA dysfunction observed in the hSYN(A53T) mice	GFAP-Nrf2/hSYN(A53T) mice strain	Gan et al. (2012)
		hDJ-1	Human DJ-1 in astrocytes protects from rotenone-induced neurodegeneration	hDJ-1 vector delivery to the substantia nigra and striatum of adult Lewis rats	De Miranda et al. (2018)
		HDAC6	Tubastatin A reduces astrocyte reactivity and neurodegeneration	Wilstar rat α-syn model	Francelle et al. (2020)
AD	CMA substrate	Tau	Tau degradation by CMA. Effect of mutant forms in CMA performance	Murine neuroblastoma cell line	Wang et al. (2009)
		β-amyloid	Oligomers tagged with KFERQ motifs enter in the lysosomes	iPSC cortical neurons derived from AD patient fibroblasts	Dou et al. (2020)
			Amyloid precursor protein (APP) contains KFERQ motif associated to its processing	Neuroblastoma cell line	Park et al. (2016)
HD	CMA inductor?	HTT	Mutant HTT induces the expression of *LAMP2A* and *HSC70*	18QHtt and 111QHtt knock-in mice. Striatum neuronal cultures from HD94 Human lymphoblast from HD patients	Koga et al. (2011a,b)
			Attachment of KFERQ motifs to HTT induces it degradation by CMA	Murine neuroblastoma cell line, R6/2 HT mice	Bauer et al. (2010)
SCA7	CMA substrate?	Ataxin-7	May be degraded by CMA and poliQ expanded ataxin-7 blocks degradation of other CMA substrates	Mouse Purkinje cells	Duncan et al. (2010)
ALS/FTLD	CMA substrate	TDP-43	TDP-43 degradation by CMA. Effect of mutant forms in CMA performance	Liver and brain of Sprague–Dawley rats	Ormeño et al. (2020)
Prion disease	CMA substrate	PrP	Downregulation of LAMP2A and HSC70. PLK3 induces degradation of PrP by CMA	263K-infected hamster and SMB-S15 cell line	Wang et al. (2017)

*PD, Parkinson’s disease; AD, Alzheimer’s disease; HD, Huntington’s disease; SCA7, spinocerebellar ataxia 7; ALS, Amyotrophic lateral sclerosis; FTLD, Frontotemporal lobal degerenation; α-syn, α-synuclein; CFS, cerebrospinal fluid; DMV, Dorsal motor nucleus of the vagus nerve*.

### Parkinson’s Disease (PD)

PD is the second most common neurodegenerative disorder after Alzheimer’s disease. It is characterized by an extensive and progressive loss of dopaminergic neurons within the substantia nigra and aggregation of the protein α-synuclein in Lewy bodies. PD is mostly sporadic, except in a 5–10% familial, and mainly related to α-synuclein and leucine-rich repeat kinase 2 (LRRK2) mutations (Campbell et al., 2018; Tolosa et al., 2020). It has been shown that α-synuclein contains a KFERQ-like motif (Cuervo et al., 2004), whereas LRRK2 contains a total of 8 putative motifs (Orenstein et al., 2013). These pieces of evidence are accompanied by both *in vitro* and *in vivo* studies demonstrating that they can be degraded by CMA (Cuervo et al., 2004; Mak et al., 2010; Orenstein et al., 2013). Interestingly, this motif is masked in the structure of α-synuclein fibers, supporting the evidence that, once in an oligomeric state, the protein is no longer available for CMA degradation (Kirchner et al., 2019). Indeed, ubiquitylation in ^96^K and protein partners binding KFERQ-like motif may prevent degradation by CMA of free soluble α-synuclein (Kirchner et al., 2019). In the same line, pathogenic mutant or even dopamine-modified species of α-synuclein and LRRK2 are not able to be degraded by CMA but present a significantly higher affinity to bind to LAMP2A protein than other CMA substrates, blocking the degradation of the later ones (Cuervo et al., 2004; Martinez-Vicente et al., 2008; Orenstein et al., 2013). These data could suggest that changes in the KFERQ-like motif sequence or even any post-translational modification modifying it may be responsible for this augmented affinity of pathogenic forms to reach the lysosomal membrane. Currently, there is not any evidence regarding this idea, so future studies should be intended to determine its impact on CMA disruption in PD.

A familial PD-associated mutation in ubiquitin C-terminal hydrolase L1 (UCH-L1) has been also described as an inhibitor of CMA activity, by promoting an induced aberrant interaction with LAMP2A, HSC70, and HSP90 (Kabuta et al., 2008). Conversely, studies regarding alterations in *de novo* LAMP2A expression regulation have been also performed. In this line, mutations in *LAMP2* gene promoter itself have been also associated with sporadic PD patients (Pang et al., 2012), and there is a decrease in LAMP2A and HSC70 expression in the substantia nigra and amygdala of PD brains compared to control samples (Alvarez-Erviti et al., 2010). Moreover, inductors of PD animal models such as 6-hydroxydopamine (6-OHDA) and rotenone, or even methamphetamine exposure, alter the expression of both Lamp2a and Hsc70, leading to neurotoxicity caused by α-synuclein accumulation (Gao et al., 2014; Sala et al., 2016; Wang et al., 2018b; Sun et al., 2019). Interestingly, several studies have also identified a set of miRNAs that regulate negatively LAMP2A and HSC70 in PD models (Alvarez-Erviti et al., 2013; Li et al., 2014).

In addition to expression alterations, impairment on LAMP2A trafficking has been also found in PD. In particular, it has been suggested that the impaired endosome-to-Golgi retrieval of Lamp2a in VPS35-deficient dopaminergic neurons may contribute to the accumulation of α-synuclein (Tang et al., 2015).

On the contrary, re-activation of CMA by upregulating *lamp2a* in neurons ameliorate α-synuclein-induced dopaminergic neurodegeneration in mice (Xilouri et al., 2013). Similarly, overexpression of CMA regulators such as *nrf2* (Gan et al., 2012) or hDJ-1 in astrocytes (De Miranda et al., 2018) presented also a decreased α-synuclein accumulation. These studies reinforce the role of CMA in PD and highlight the benefits of CMA restoration.

The idea of using CMA effectors as biomarkers for the diagnosis of PD has been also investigated. Indeed, it has been shown that LAMP2 concentrations are decreased in female LRRK2 PD patients compared to healthy individuals (Klaver et al., 2018) in CSF samples. Besides, a significant reduction of HSC70 has been identified in peripheral blood mononuclear cells (PBMCs) of patients of sporadic PD, but no differences in LAMP2A were detected (Sala et al., 2014). Further research should be needed to explore in more detail the potential role of CMA as a PD trait biomarker.

Pharmacological activation of CMA has been postulated as a therapeutic strategy (Campbell et al., 2018). In support of this, co-culture of human pluripotent stem cell (iPSC)-derived astrocytes and neurons from control and PD patients, showed that chemical enhancement of CMA protected both from degeneration (di Domenico et al., 2019). In line with this, the treatment with the selective inhibitor of HDAC6 Tubastatin A reduces both astrocyte reactivity and neuron degeneration induced by α-synuclein in a rat PD model, increasing Lamp2a and Hsc70 expression and partially inducing α-synuclein acetylation (Francelle et al., 2020). Crosstalk between different protein degradation systems is also noteworthy in PD pathogenesis. It has been described that not only CMA but also UPS can participate in the degradation of soluble α-synuclein (Webb et al., 2003). As protein aggregation occurs, macroautophagy is activated as a secondary mechanism to compensate for CMA/UPS blockage and alleviate these conditions (Webb et al., 2003). Interestingly, the connection between autophagy and UPS is not limited to the removal of cytosolic proteins but also involves the removal of organelles. In this line, it has been described that ubiquitination of constituent proteins in the membrane of peroxisomes mediates their macroautophagy, whereas the familial form of PD-associated ubiquitin ligase parkin ubiquitinates mitochondrial proteins, inducing mitophagy (Wong and Cuervo, 2010).

### Alzheimer’s Disease

Alzheimer’s disease (AD) is the most common form of dementia in elderly people, characterized classically by the presence of β-amyloid plaques and neurofibrillary tangles of hyperphosphorylated and cleaved forms of the microtubule-associated protein tau (Weller and Budson, 2018). In AD, the translocated mutant tau products at the lysosomal membrane hamper the binding and translocation of other CMA substrates, decreasing their activity (Wang et al., 2009). Not only this, but reduced targeting to the lysosomes has been also observed in a mutant form of tau (Caballero et al., 2018), corroborating a general impaired CMA activity in AD. Interestingly, a regulator of calcineurin 1 (RCAN1) is elevated in AD and contains KFERQ motifs that habilitate it to be degraded by CMA (Liu et al., 2009). As RCAN1 is an inhibitor of calcineurin-dependent dephosphorylation of tau protein, it would be worthy to test the hypothesis that increased degradation of RCAN1 by activation of CMA could be mediating, at least in part, the reduction of the formation of tau aggregates. Together with this, AD-associated ubiquilin is degraded by CMA (Rothenberg et al., 2010), reinforcing the impact of this type of autophagy in AD pathogenesis. Interestingly, a recent study showed that tagging β-amyloid oligomers with multiple KFERQ motifs promoted their entering in endosomes and lysosomes, protecting human primary cultured cortical neurons from neurotoxicity (Dou et al., 2020). Indeed, amyloid precursor protein (APP) by itself, where β-amyloid is derived from, contains a KFERQ motif at its C-terminus which, intriguingly, has been associated with the correct APP processing, not to CMA-dependent degradation (Park et al., 2016). However, deletion of this motif increases the amount of APP cleaved products, suggesting that other KFERQ motif-dependent protein degradation system may be responsible for this effect.

### Huntington’s Disease

Huntington’s disease (HD) is a dominantly inherited pathology caused by the accumulation of mutant huntingtin protein (HTT) that contains an expanded N-terminal polyglutamine (polyQ) tract (Bauer et al., 2010). HD is the only neurodegenerative disease with elevated LAMP2A and HSC70 expression (Koga et al., 2011a), as a result of both UPS and macroautophagy failure. The use of an adaptor that contains two copies of polyQ binding sequences and two different KFERQ motifs, directs specifically mutant HTT to the CMA machinery for its degradation, ameliorating symptoms, and extending the life span of an HD mouse model (Bauer et al., 2010). However, the efficiency of this compensatory mechanism may not be sufficient to control the evolution of the disease (Kon et al., 2011), due to the age-dependent CMA activity decline. Thus, the effect of high levels of CMA regulators and whether pharmacological activation of CMA could be beneficial in the evolution of the disease should be investigated.

### Other Neurodegenerative Disorders

Associations between CMA alterations and other neurodegenerative disorders have been also described in the last few years. On the one hand, some studies showed that both wild-type and pathological forms of TAR DNA binding protein 43 kDa (TDP-43), whose accumulation in neurons is associated with amyotrophic lateral sclerosis and frontotemporal lobar degeneration, are substrates of CMA and can affect this degradative system (Ormeño et al., 2020). Interestingly, macroautophagy and endocytosis connection is also important in models of frontotemporal dementia. Impaired formation of multivesicular bodies due to ESCRT-III dysfunction in the membrane of the late endosomes reduces autophagy activity and induces autophagosome accumulation (Wong and Cuervo, 2010). This idea demonstrates the crosstalk between multiple types of lysosomal processes in this neurodegenerative disorder.

On the other hand, animal models for mutant prion protein (PrP) disease present downregulation of LAMP2A and HSC70 proteins, and that the overexpression of polo-like kinase 3 (PLK3) induces PrP degradation by CMA (Wang et al., 2017).

Overall, either by the negative effect of the mutant proteins or by the reduction on the expression of the machinery itself, a downregulation of CMA has been described in most neurodegenerative diseases. Further research directed to the study of the other regulatory levels of CMA discussed in previous sections in this work would contribute to the comprehensive view of the altered physiological mechanisms leading to CMA downregulation in ND (Figure 3). Together with this, the potential of CMA as a diagnosis/prognosis biomarker and its use as a therapeutic target remains to be elucidated.

## Cma in Glioblastoma

Glioblastoma (GBM) represents the most common and aggressive primary brain tumor and it is classified as grade IV glioma, by the World Health Organization (WHO; Louis et al., 2016). Recent pieces of evidence support that CMA is hyper-activated in GBM (Table 3). Moreover, surgical samples from patients treated with temozolomide (TMZ) chemotherapy present induced expression of LC3B, LAMP1, and LAMP2A compared to the initial sample (Natsumeda et al., 2011). Curcumin treatment, which has shown anti-tumor effects in GBM, downregulates the expression of LAMP2A in GBM cell lines (Maiti et al., 2019) suggesting a cell-autonomous effect of CMA in GBM. In this direction, GBM-associated proteins such as TP53, EGFR, or HIF1α have been identified as CMA substrates (Vakifahmetoglu-Norberg et al., 2013). However, the implication of their altered CMA-mediated degradation in GBM remains to be elucidated. A quiescent subset of endogenous glioma cells named glioma stem cells (GSC) is responsible for sustaining long-term tumor growth and therapy resistance (Chen et al., 2012). The impact of macroautophagy in GSCs has been widely described (Nazio et al., 2019), but the role of CMA fully remains to be investigated. The fact that CMA substrates such as EGFR or HIF1α have been associated with GSCs population (Emlet et al., 2014), and that curcumin treatment potentially may target GSC subpopulation (Fong et al., 2010), indirectly links CMA to GSC activity. However, other studies claim that blocking CMA-mediated HIF1α degradation induces resistance to TMZ (Lo Dico et al., 2018) and that TMZ resistant cells are unable to increase cytoplasmic ROS levels and activate CMA, preventing thus GBM cell toxicity (Lo Dico et al., 2019). In this sense, it has been described that the inhibition of macroautophagy increases the susceptibility of GSCs to TMZ by augmenting ferroptosis, a CMA-associated necrotic cell death mechanism (Buccarelli et al., 2018). Thus, further studies are needed to elucidate the role of CMA in GSC subpopulation and therapy resistance.

**Table 3:** Main studies revealing CMA upregulation in glioblastoma (GBM).

Target	Role in CMA	Finding	Model of study	Reference
LAMP2A	Key effector	CMA defective pericytes reduce proliferation of GB.	Murine primary pericytes and U373/U87 GB cell lines.	Valdor et al. (2019)
		Curcumin reduces LAMP2A expression.	U-87MG, GL261, F98, C6-glioma, and N2a cells.	Maiti et al. (2019)
		TMZ treatment induce expression of LAMP2A	pre-/post-TMZ treatment human tumors.	Natsumeda et al. (2011)
		Block of CMA-mediated HIF1α degradation induces resistance to TMZ.	U251, U87, T98, U138 GB cell lines.	Lo Dico et al. (2018)
		TMZ resistant cells are unable to increase ROS levels and activate CMA, preventing GB cell toxicity	U251 and T98 cell lines.	Lo Dico et al. (2019)
Ferroptosis	Process in which CMA participate.	Macroautophagy inhibition increases susceptibility of GSCs to TMZ by inducing ferroptosis	Patient-derived GSCs and deparaffinized human tumor sections.	Buccarelli et al. (2018)

Interestingly, a GBM mouse model grafted *in vivo* with CMA-defective pericytes shows reduced GB proliferation and effective immune response compared to the ones grafted with control pericytes (Valdor et al., 2019). This fact, together with other studies associating CMA to tumor-associated macrophages in other types of cancer (Guo et al., 2017; Wang et al., 2019b), highlights the potential role of CMA in the crosstalk between the tumor cells and the microenvironment.

Overall, studies regarding CMA in GBM have focused on the impact of the modulation of LAMP2A expression by itself (Figure 3). Nevertheless, the status of LAMP2A trafficking or stability in the lysosomal membrane or even post-translational modifications of LAMP2A, HSC70, or even KFERQ-like motifs of CMA substrates has not been explored.

Of note, it is known that GBM cells need to maintain balanced proteostasis for their survival and progression, by complex crosstalk between protein synthesis, unfolded protein response, stress response pathways, autophagy, and UPS (Yang et al., 2019). However, future studies will be necessary to assess the possible association of CMA with other protein degradation systems in GBM, as it is still a young field and no evidence has been published so far.

## Therapeutic Targeting of Cma

The impact of autophagy as a therapeutic target in human brain pathologies has been studied mainly in terms of macroautophagy, where many preclinical studies in PD and GBM have been developed (Towers and Thorburn, 2016; Moors et al., 2017; Taylor et al., 2018). Indeed, few initial clinical trials in GBM with the lysosomal inhibitor chloroquine have been also performed, but with no significant overall survival improvement due to dose-limiting toxicity (Sotelo et al., 2006; Rosenfeld et al., 2014). Importantly, macroautophagy modulators such as 3-methyladenine, LY294002, vinblastine, or rapamycin do not alter CMA activity (Finn et al., 2005). Although to date, there are not specific modulators of CMA, several compounds have demonstrated to alter key effectors of this process. On the one hand, compounds such as geldanamycin (Pedrozo et al., 2013), 6-aminonicotinamide (Finn et al., 2005), glucose-6-phosphate dehydrogenase inhibitor (Finn et al., 2005), silymarin (Tripathi et al., 2020), chronic caffeine (Luan et al., 2018), manganese (Yan et al., 2019), trehalose (Rusmini et al., 2019), β-Asarone (Huang et al., 2016) and other compounds extracted from natural medicinal plants (Wu et al., 2019a), or even combination treatments with bortezomib and suberoylanilide hydroxamic acid (SAHA; Watanabe et al., 2010) increase LAMP2A levels and thus, CMA activity. This therapeutic strategy could be beneficial for neurodegenerative diseases, where, as mentioned before, CMA activity is downregulated.

On the other hand, treatment with compounds such as P140 (Macri et al., 2015), vitamin E (Cao et al., 2009), protein synthesis inhibitors anisomycin and cycloheximide (Finn et al., 2005) or p38MAPK inhibitor (Finn et al., 2005) downregulate CMA, a strategy that may be beneficial for cancer context. Overall, as mentioned before, the modulatory effects of all these compounds are not specific for CMA machinery, they have many other targets. Thus, novel strategies must be developed to get new insights regarding CMA-specific and thus, safer therapeutic approaches. For this, many advances have been done regarding both *in vitro* and *in vivo* models for the study of CMA (Juste and Cuervo, 2019). Among them, *in vitro* methods based on the use of a photoconvertible fluorescent reporter (Koga et al., 2011b) or a “GAPDH-HaloTag” fusion protein (Seki et al., 2012; Sato et al., 2016), and a recently described transgenic reporter mouse that allows dynamic measurement of CMA activity *in vivo* (Dong et al., 2020) will facilitate further advances in the field.

## Concluding Remarks and Future Perspectives

CMA is a protein selective type of autophagy with increasing evidence revealing a role in homeostasis and pathology of a wide variety of organs, with special emphasis on aging and cancer. Regarding the brain, and in line with the protective role of CMA against protein damage and formation of toxic aggregates, much of the evidence gathered so far is focused on neurodegenerative diseases. Alterations in the expression of CMA regulators and the blocking effect of mutant CMA substrates show the reduced activity of this process in disorders such as PD or AD. The study of CMA in physiological brain aging is still a relatively young field, but recent pieces of evidence support the hypothesis that CMA could be involved in the decline in brain homeostasis and regeneration capacity. To consolidate this idea, future studies should be directed to understanding the impact of CMA in different cell types, with special interest on the neurogenic niches, whose impairment has been linked to age-associated cognitive and functional decline. Intriguingly, some evidence demonstrates that glioblastoma presents an upregulated activity of CMA, not only affecting tumor cells but also their crosstalk with pericytes. Similarly, recent studies in PD also show a CMA-mediated modulation of the crosstalk between astrocytes and neurons, highlighting its promising role in intercellular communication. Thus, further efforts should be done to elucidate the impact of CMA in the brain microenvironment during physiological and pathological aging.

Overall, CMA alterations in brain aging, neurodegenerative diseases, or glioblastoma could be interpreted in two directions. On the one hand, it could be postulated that CMA-mediated degradation of key disease-relevant proteins can be modulated, so that while aged cells are not able to degrade mutant species of physiological CMA substrates, cancer cells use CMA to eliminate proteins such as tumor suppressor. On the contrary, it may be suggested that it is the entire CMA process that is altered, being downregulated in aging and upregulated in glioblastoma progression. These ideas encourage further research to understand the specific mechanisms altering CMA activity in brain aging and glioblastoma, as well as to develop selective modulators of CMA as new strategies against age-related pathologies.

## Author Contributions

JA-I wrote the draft and AM reviewed it and supervised the work.

## Conflict of Interest

The authors declare that the research was conducted in the absence of any commercial or financial relationships that could be construed as a potential conflict of interest.
